# Effectiveness of *Yushen Hezhi* therapy for postmenopausal osteoporosis: An overview of systematic reviews of randomized controlled trials

**DOI:** 10.3389/fendo.2022.1015483

**Published:** 2022-09-26

**Authors:** Jinlong Zhao, Xiao Xiao, Guanghui Zhou, Nanjun Xu, Jun Liu

**Affiliations:** ^1^ The Second Clinical Medical College of Guangzhou University of Chinese Medicine, Guangzhou, China; ^2^ The Second Affiliated Hospital of Guangzhou University of Chinese Medicine, Guangzhou, China; ^3^ The Research Team on Bone and Joint Degeneration and Injury of Guangdong Provincial Academy of Chinese Medical Sciences, Guangzhou, China; ^4^ The Fifth Clinical Medical College of Guangzhou University of Chinese Medicine, Guangzhou, China; ^5^ Guangdong Second Traditional Chinese Medicine Hospital (Guangdong Province Enginering Technology Research Institute of Traditional Chinese Medicine), Guangzhou, China

**Keywords:** Chinese traditional medicine, Yushen Hezhi therapy, postmenopausal osteoporosis, systematic review, Chinese medicine

## Abstract

**Objective:**

To review systematic reviews (SRs) and meta-analyses (MAs) of *Yushen Hezhi* therapy (YSHZT) for postmenopausal osteoporosis (PMOP) to provide an evidence-based recommendation for researchers and decision makers.

**Methods:**

We searched the PubMed, Cochrane Library, Embase, China National Knowledge Infrastructure (CNKI), China Biology Medicine (CBM) and Wanfang databases for published SRs and MAs on YSHZT for the treatment of PMOP. The retrieval time was limited to July 2022. The Assessing the Methodological Quality of Systematic Reviews (AMSTAR)-2 tool and Grades of Recommendations, Assessment, Development, and Evaluation (GRADE) classification system were used to evaluate the methodological quality and the evidence quality of the SRs and MAs, respectively.

**Results:**

A total of 14 SRs and MAs involving 14720 cases of PMOP were included. The results of the methodological quality evaluation indicated that there were no studies with medium- or high-quality methodology included in the study and that there were 9 and 5 low- and very low-quality studies, respectively. The GRADE evaluation results show that while there was no high-level evidence based on 86 evaluation indicators, there was 1 study with moderate-level evidence (1%), 44 studies with low-level evidence (51%) and 41 with very low-level evidence (48%) based on other indicators. YSHZT can significantly improve the bone mineral density (BMD) of Ward’s triangle, with a mean difference range of 0.03 to 0.12. Different conclusions were reported regarding the BMD of the lumbar spine, femoral trochanter, femoral neck, and hip, as well as bone turnover markers, adverse reactions and other outcome indicators in different SRs and thus still need further study.

**Conclusions:**

The methodological quality and the evidence quality of the outcome indicators for YSHZT in the treatment of PMOP are poor, and the efficacy and safety of YSHZT in the treatment of PMOP still need to be further verified by more high-quality studies.

## 1 Introduction

Osteoporosis (OP) is a common disease that is characterized by decreased bone mass and decreased bone strength and is associated with an increased risk of brittle fracture (1). Postmenopausal women are particularly prone to OP because of ovarian ageing or oestrogen deficiency, which leads to bone metabolism imbalance and bone loss. In addition, the complications caused by OP can seriously impair the health and decrease the quality of life of postmenopausal women (2, 3). The incidence of fractures varies widely, but an average of up to 50% of women older than the age of 50 are at risk of fracture (4). There is no doubt that postmenopausal osteoporosis (PMOP) is a public health problem that requires attention. Therefore, the multidisciplinary management of PMOP, including nursing, drug therapy, surgery, physical activity and rehabilitation, plays an important role in improving the quality of life of patients and reducing the corresponding economic burden (5–7).

At present, drugs for the treatment of PMOP include oestrogen, alendronate sodium, calcitonin, fluoride, and parathyroid hormone, and although these drugs have definite therapeutic effects, it is undeniable that long-term use of these drugs can be accompanied by nonnegligible adverse reactions, such as gastrointestinal discomfort, cardiovascular risk, atypical fracture and jaw bone necrosis (8–10). Therefore, there is an urgent need for additional treatment methods to be explored. Traditional Chinese medicine (TCM) approaches have certain characteristics and advantages regarding the treatment of PMOP and can play an anti-OP role through multiple channels and targets (11, 12). In TCM, OP belongs to the categories of *Gubi* and *Guwei*. TCM treatments for PMOP include acupuncture, massage, prescriptions, and Chinese patent medicine, and these treatments are considered to play potential roles in promoting the dynamic balance of osteoblasts and osteoclasts (13–15). New researchers studying TCM have found that kidney deficiency and blood stasis are the main pathogenic factors of OP and hypothesize that *Yushen Hezhi* therapy (YSHZT) can achieve good curative effects in patients receiving treatment for OP (16). YSHZT is a method summarized by Liu Jun for treating orthopaedic diseases according to TCM theory, and it suggests that kidney deficiency and blood stasis are responsible for the pathogenesis of OP and knee osteoarthritis (17). TCM prescriptions or Chinese patent medicines based on YSHZT include Duhuo Jisheng decoction, Bushen Huoxue decoction, Xianling Gubao capsule, Zuogui pill, and Liuwei Dihuang pill, among others (18–22). In recent years, there has been ongoing research on the use of YSHZT in TCM for tonifying the kidneys and activating blood circulation, and many researchers have published related clinical studies, systematic reviews (SRs) and meta-analyses (MAs). However, among SRs, conclusions regarding the treatment of PMOP using YSHZT are not completely consistent; only high-quality SRs have positive guiding value, and studies with low-quality evidence-based recommendations can reach misleading conclusions (23). This study collected SRs and MAs on the treatment of PMOP based on the use of YSHZT and used methodological quality evaluation tools and an evidence level evaluation system to evaluate the methodological quality of the studies and the reliability of the conclusions to provide a reference for evidence users.

## 2 Materials and methods

This study was reviewed according to the Cochrane Handbook for Systematic Reviews of Interventions and reported according to the Preferred Reporting Items for Systematic Reviews and Meta-Analyses (PRISMA) statement (24, 25).

### 2.1 Inclusion criteria

1) Study type: SRs or MAs based on randomized controlled studies (RCTs). 2) Study subjects: PMOP patients with no limitations on age, sex, race, nationality or disease course. 3) Intervention measures: treatment with a TCM decoction and Chinese patent medicine under the guidance of YSHZT, such as Bushen Huoxue decoction, Duhuo Jisheng decoction, or Liuwei Dihuang pill, in the experimental group (EG). 4) Controls: treatment with routine drug therapy, a blank control or placebo in the control group (CG). 5) Outcome indicators: bone mineral density (BMD), alkaline phosphatase (ALP), oestradiol (E2), serum Ca (S-Ca), serum phosphorus (S-P), clinical effective rate, visual analysis scale (VAS) score, and adverse events (AEs), among others.

### 2.2 Exclusion criteria

1) Incomplete data or unavailable full text; 2) duplicated publication; 3) network MA; 4) SR or MA protocols; and 5) same therapy as in the EG intervention included in the control.

### 2.3 Retrieval strategy

We searched the Cochrane Library, PubMed, Embase, China National Knowledge Infrastructure (CNKI), China Biology Medicine (CBM) and Wanfang databases for SRs and MAs published from the establishment of each database to July 2022. A combination of MeSH and free words, including Bushen Huoxue therapy, tonifying kidney, Bushen Huoxue, Bushen Huayu, Chinese medicine, Chinese herb, osteoporosis, postmenopausal osteoporosis, systematic review and meta-analysis, were used. The retrieval strategy for each database is shown in **Supplementary Material 1**.

### 2.4 Literature screening and data extraction

Two researchers screened and checked the search results, conducted two rounds of screening by reading the title, abstract and full text, and finally determined the included SRs or MAs according to the inclusion and exclusion criteria. The two researchers extracted and cross-checked the information and data included in the literature. The specific contents extracted included author, year of publication, age, sample size, intervention measures, quality evaluation methods, and outcome indicators.

### 2.5 Methodological evaluation

We used the Assessing the Methodological Quality of Systematic Reviews (AMSTAR)-2 tool to evaluate the quality of the methodology of the included studies and grade the results (26). The AMSTAR-2 tool contains 16 items, including 7 key items. We classified each document as having high, moderate, low or very low reliability. The classification criteria were as follows: high (noncritical item defect ≤ 1); moderate (noncritical item defect>1); low (critical item defect=1, with or without noncritical item defect); and very low (critical item defect>1, with or without noncritical item defect).

### 2.6 Evaluation of evidence quality

The Grades of Recommendations, Assessment, Development, and Evaluation (GRADE) classification system was used to evaluate the evidence level of the included literature outcome indicators (27, 28). The degradation factors of the GRADE system include limitations, indirectness, inconsistency, inaccuracy and publication bias, and the level of evidence is evaluated according to the following criteria: if there are no degradation factors, it will be rated as high quality; if there are one or two degradation factors, it will be rated as moderate and low quality, respectively; and if there are three or more degradation factors, it will be rated as critically low quality.

### 2.7 Statistical analysis

We used descriptive analysis to summarize the evidence results of the included SRs. Based on each outcome index, the efficacy and safety of YSHZT in the treatment of PMOP were re-evaluated.

## 3 Results

### 3.1 Literature screening results and characteristics of the included studies

**Figure 1:**
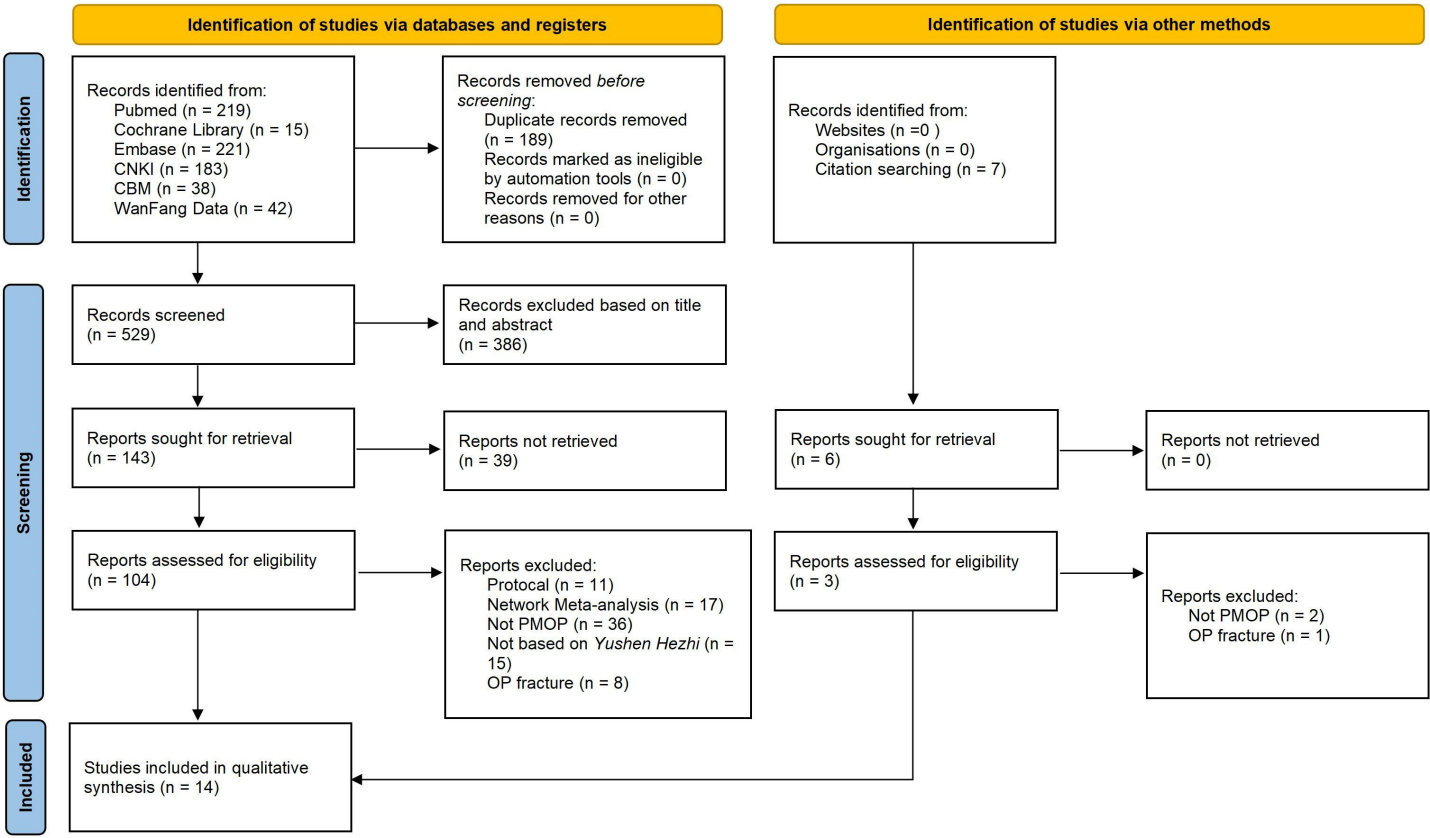
Flow diagram of literature screen process.

We preliminarily retrieved 529 studies; after further reading of the titles and abstracts, 386 irrelevant records were excluded. After reading the full text with reference to the inclusion and exclusion criteria in combination with screening of the references included in the study, 14 (29–42) SRs or MAs were ultimately included. The list of of studies excluded after reading the full text and reasons are shown in **Supplementary Material 3**. The literature screening process and results are shown in **Figure 1**.

A total of 14720 patients were included in the 14 studies (29–42). All included patients were postmenopausal women who were diagnosed with osteoporosis, not including secondary osteoporosis. The intervention measures in the EG based on YSHZT included Bushen Huoxue decoction, tonifying liver-kidney decoction, Duhuo Jisheng decoction, and Xianling Gubao capsule, among others, while conventional drugs or a placebo were applied in the CG. All the included studies (29–42) used the Cochrane risk-of-bias assessment tool for quality assessment, and three of them (30, 31, 39) also applied the Jadad score. Only one study (29) used the GRADE system to evaluate the level of evidence quality. The basic characteristics of the included literature are shown in **Table 1**.

**Table 1:** Characteristics of the systematic reviews or meta-analyses included in the overview.

Study	Number of RCTs	Number of participants		Intervention		Methodological quality evaluation tool	GRADE evaluation	Outcomes assessed
		EG	CG	EG	CG			
Li J 2020 (29)	7	350	350	DHJSD (YSHZT) (or plus CG therapy)	No treatment, placebo, or conventional therapy	ROB	Yes	Clinical Effective Rate, LBMD, BGP, E2, AE
Chen T 2020 (30)	19	764	716	Tonifying liver-kidney decoction (YSHZT) (or plus CG therapy)	Conventional drug therapy	ROB and Jadad scale	No	Clinical Effective Rate, LBMD, VAS, BGP, ALP, S-Ca, E2
Zhao S 2019 (31)	18	708	700	TCM Compound Preparation for Tonifying Kidney and Activating Blood Circulation (YSHZT) (or plus CG therapy)	Conventional drug therapy	ROB and Jadad scale	No	Clinical Effective Rate, LBMD, FNBMD, VAS, ALP, S-Ca, S-P, E2, IL-6, AE
Chen P 2021 (32)	11	592	589	Bushen Huoxue Decoction (YSHZT) (or plus CG therapy)	Conventional drug therapy	ROB	No	Clinical Effective Rate, LBMD, VAS, BGP, AE
Ma X 2022 (33)	14	625	593	Method of invigorating kidney, invigorating spleen and promoting blood circulation (YSHZT) (or plus CG therapy)	Conventional drug therapy	ROB	No	Clinical Effective Rate, LBMD, FNBMD, ALP, E2, IL-6
Cui X 2019 (34)	7	244	247	Method of invigorating kidney and promoting blood circulation (YSHZT) (or plus CG therapy)	Conventional drug therapy	ROB	No	Clinical Effective Rate, LBMD, VAS
Cai X 2022 (35)	13	653	653	DHJSD (YSHZT) (or plus CG therapy)	Conventional drug therapy	ROB	No	Clinical Effective Rate, LBMD, FNBMD, HBMD, BGP, ALP, S-Ca, S-P, E2, AE
Jing Y 2021 (36)	14	713	73	DHJSD (YSHZT) (or plus CG therapy)	Conventional drug therapy	ROB	No	Clinical Effective Rate, LBMD, HBMD, E2, S-Ca
Zhang Y 2021 (37)	12	535	525	Erxian Decoction (YSHZT) (or plus CG therapy)	Conventional drug therapy	ROB	No	Clinical Effective Rate, LBMD, FNBMD, FTBMD, Ward’s triangle BMD, BGP, ALP, S-Ca, S-P, E2, AE
Wang Y 2020 (38)	12	616	612	Liuwei Dihuang pill (YSHZT) (or plus CG therapy)	Conventional drug therapy	ROB	No	Clinical Effective Rate, LBMD, FNBMD, HBMD, VAS, AE
Liu H 2020 (39)	10	403	402	QingE Wan (YSHZT) (or plus CG therapy)	Conventional drug therapy	ROB and Jadad scale	No	LBMD, FNBMD, FTBMD, Ward’s triangle BMD, VAS, AE
Chen F 2021 (40)	15	470	445	QingE Wan (YSHZT) (or plus CG therapy)	Conventional drug therapy	ROB	No	Clinical Effective Rate, LBMD, FNBMD, FTBMD, Ward’s triangle BMD, VAS, E2
Xu G 2021 (41)	11	365	365	XLGB (YSHZT) (or plus CG therapy)	Ca or Calcitriol	ROB	No	Clinical Effective Rate, LBMD, FNBMD, FTBMD, AE
An Y 2019 (42)	16	754	738	XLGB (YSHZT) (or plus CG therapy)	No treatment, placebo, or conventional therapy	ROB	No	Clinical Effective Rate, LBMD, HBMD, Ward’s triangle BMD, VAS, BGP, ALP, S-Ca, S-P, E2, IL-6, AE

EG, Experimental Group; CG, Control Group; YSHZT, Yushen Hezhi therapy; DHJSD, Duhuo Jisheng Decoction; XLGB, Xianling Gubao capsule; ROB, Cochrane Risk of Bias Tool; ALP, Alkaline Phosphatase; E2, Estradiol; S-Ca, Serum Ca; S-P, Serum Phosphorus; BGP, Bone Gla Protein; VAS, Visual Analogue Scale; AE, Adverse Event; BMD, Bone Mineral Density; LBMD, BMD of Lumbar Spine; FTBMD, BMD of Femoral Trochanter; FNBMD, BMD of Femoral Neck; HBMD, BMD of Hip.

### 3.2 Methodological quality evaluation results

**Figure 2:**
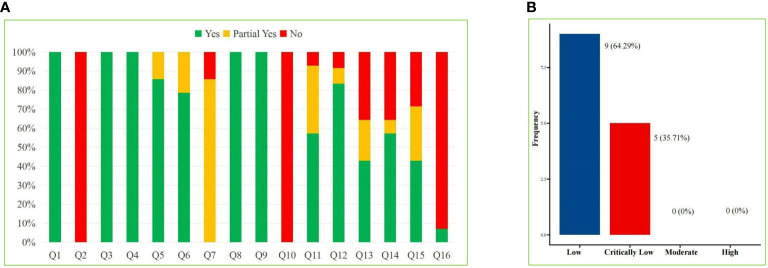
AMSTAR-2 Score Results. **(A)** Stacked bar chart of 16 items and **(B)** Straight bar graph of evidence proportion.

We applied the AMSTAR-2 tool to evaluate the methodological quality of the included SRs/MAs, and the results are shown in **Table 2** and **Figure 2**. The results showed that in the evaluation of key items, none of the included studies (29–42) met the requirement of item 2, indicating that all included studies were not registered or provided with preliminary design schemes. There were two SRs (36, 37) that do not provide a list of the criteria used for the inclusion and exclusion of literature (item 7). One study (30) did not use the effect volume consolidation method correctly (item 11). Five studies (30, 36–38, 42) did not correctly evaluate the impact of publication bias on the results (item 13). Four studies (30, 36, 37, 42) did not meet the requirements of item 15 to fully investigate the source of publication bias. All the SRs/MAs met the requirements of key items 4 and 9. The evaluation results of the above items and noncritical items are shown in **Figure 2A**. According to the AMSTAR-2 methodological quality evaluation index, there were no studies of moderate- or high-quality methodology in the 14 SRs/MAs included in this study, while there were 9 (29, 31–35, 39–41) and 5 (30, 36–38, 42) studies of low and very low quality, respectively. The overall quality grade results are shown in **Figure 2B**.

**Table 2:** AMSTAR scoring results of the included systematic reviews or meta-analysis.

Study	Q1	Q2^※^	Q3	Q4^※^	Q5	Q6	Q7^※^	Q8	Q9^※^	Q10	Q11^※^	Q12	Q13^※^	Q14	Q15^※^	Q16	Ranking of quality
Li 2020 J (29)	Y	N	Y	Y	Y	Y	PY	Y	Y	N	PY	PY	Y	Y	Y	Y	Low
Chen T 2020 (30)	Y	N	Y	Y	Y	PY	PY	Y	Y	N	N	Y	N	N	N	N	Critically Low
Zhao S 2019 (31)	Y	N	Y	Y	Y	Y	PY	Y	Y	N	Y	Y	PY	N	Y	N	Low
Chen P 2021 (32)	Y	N	Y	Y	Y	PY	PY	Y	Y	N	Y	Y	Y	N	Y	N	Low
Ma X 2022 (33)	Y	N	Y	Y	Y	Y	PY	Y	Y	N	PY	Y	Y	Y	Y	N	Low
Cui X 2019 (34)	Y	N	Y	Y	Y	Y	PY	Y	Y	N	Y	N	Y	Y	PY	N	Low
Cai X 2022 (35)	Y	N	Y	Y	Y	Y	PY	Y	Y	N	PY	Y	Y	Y	Y	N	Low
Jing Y 2021 (36)	Y	N	Y	Y	PY	PY	N	Y	Y	N	Y	Y	N	Y	N	N	Critically Low
Zhang Y 2021 (37)	Y	N	Y	Y	PY	Y	N	Y	Y	N	Y	Y	N	Y	N	N	Critically Low
Wang Y 2020 (38)	Y	N	Y	Y	Y	Y	PY	Y	Y	N	Y	Y	N	Y	Y	N	Critically Low
Liu H 2020 (39)	Y	N	Y	Y	Y	Y	PY	Y	Y	N	PY	Y	PY	PY	PY	N	Low
Chen F 2021 (40)	Y	N	Y	Y	Y	Y	PY	Y	Y	N	Y	Y	Y	N	PY	N	Low
Xu G 2021 (41)	Y	N	Y	Y	Y	Y	PY	Y	Y	N	Y	Y	PY	N	PY	N	Low
An Y 2019 (42)	Y	N	Y	Y	Y	Y	PY	Y	Y	N	PY	Y	N	Y	N	N	Critically Low

^※^key entry; PY, Partial Yes; Y, Yes; N, No. The specific contents of 16 items are shown in **Supplementary Material 2**.

### 3.3 Evidence quality grading results

The 14 SRs or MAs included 86 studies involving 14 outcome indicators. The GRADE evaluation results showed no studies with high-level evidence, 1 study with moderate-level evidence (1%), 44 studies with low-level evidence (51%) and 41 studies with very low-level evidence (48%). The results are shown in **Table 3** and **Table 4**.

**Table 3:** GRADE grading results of the included systematic reviews or meta-analysis.

Outcomes	Study	Efect Size (95% CI)	*P*	I^2^ (%)	Risk of bias	Inconsistency	Indirectness	Imprecision	Publication bias	GRADE quality
LBMD	Li 2020 J (29)	MD: 0.46 (0.24, 0.68)	< 0.001	21	Serious	No	No	No	Serious	Low
	Chen T 2020 (30)	MD: 0.12 (0. 07, 0. 17)	< 0.01	98	Serious	Serious	No	No	No	Low
	Zhao S 2019 (31)	SMD: 0.19 (0.06, 0.33)	0.005	0	Serious	No	No	No	Serious	Low
	Chen P 2021 (32)	SMD: 1.22 >(0.62, 1.81)	< 0.001	95	Serious	Serious	No	Serious	Serious	Very low
	Ma X 2022 (33)	MD: 0. 01 (-0.02, 0.04)	0.42	88	Serious	Serious	No	No	Serious	Very low
	Cui X 2019 (34)	MD: 0.01 (-0.01, 0.03)	0.43	41	Serious	No	No	No	Serious	Low
	Cai X 2022 (35)	MD: 0.36 (0.24, 0.49)	< 0.001	99	Serious	Serious	No	No	Serious	Very low
	Jing Y 2021 (36)	MD: -0. 02 (-0.06, 0.02)	0.30	0	Serious	No	No	No	Serious	Low
	Zhang Y 2021 (37)	MD: 0.88 (0.70, 1.06)	< 0.001	40	Serious	No	No	Serious	Serious	Very low
	Wang Y 2020 (38)	MD: 0.06 (-0.06, 0.18)	0.31	0	Serious	No	No	No	Serious	Low
	Liu H 2020 (39)	MD: 0.05 (0.00, 0.10)	0.05	83	Serious	Serious	No	No	Serious	Very low
	Chen F 2021 (40)	MD: 0.04 (0.02, 0.05)	< 0.001	42.9	Serious	No	No	No	Serious	Low
	Xu G 2021 (41)	SMD: 0.75 (0.37, 1.13)	< 0.001	78	Serious	Serious	No	Serious	Serious	Very low
	An Y 2019 (42)	MD: 0.08 (0.03, 0.14)	0.002	0	Serious	No	No	No	Serious	Low
FNBMD	Zhao S 2019 (31)	SMD: 0.26 (-0.05, 0.56)	0.057	35.5	Serious	No	No	No	Serious	Low
	Ma X 2022 (33)	MD: 0.00 (-0.06, 0.05)	0.91	87	Serious	Serious	No	No	Serious	Very low
	Cai X 2022 (35)	MD: 0.13, (0.09, 0.17)	< 0.001	0	Serious	No	No	No	Serious	Low
	Zhang Y 2021 (37)	SMD: 0.62 (0.44, 0.79)	< 0.001	36	Serious	No	No	No	Serious	Low
	Wang Y 2020 (38)	MD: 0.4 (0.26, 0.54)	< 0.001	0	Serious	No	No	No	Serious	Low
	Liu H 2020 (39)	MD: 0.06 (0.01, 0.11)	0.02	84	Serious	Serious	No	No	Serious	Very low
	Chen F 2021 (40)	MD: 0.06 (0.00, 0.11)	0.038	92.5	Serious	Serious	No	No	No	Low
	Xu G 2021 (41)	SMD: 0.89 (0.47, 1.32)	< 0.001	83	Serious	Serious	No	Serious	Serious	Very low
HBMD	Jing Y 2021 (36)	MD: 0.55 (0.11, 1.0)	0.02	71	Serious	Serious	No	Serious	Serious	Very low
	Wang Y 2020 (38)	MD: 0.55 (0.26, 0.84)	< 0.001	72	Serious	Serious	No	No	Serious	Very low
	An Y 2019 (42)	MD: 0.02 (-0.01, 0.05)	0.26	0	Serious	No	No	No	Serious	Low
FTBMD	Zhang Y 2021 (37)	MD: 0.03 (0.02, 0.03)	< 0.001	0	Serious	No	No	No	Serious	Low
	Liu H 2020 (39)	MD: 0.03 (0.00, 0.07)	0.07	70	Serious	Serious	No	No	Serious	Very low
	Chen F 2021 (40)	MD: 0.07 (0.04, 0.11)	< 0.001	0	Serious	No	No	No	Serious	Low
	Xu G 2021 (41)	SMD: 0.74 (0.51, 0.98)	< 0.001	33	Serious	No	No	No	Serious	Low
Ward’s triangle BMD	Zhang Y 2021 (37)	MD: 0.03 (0.02, 0.03)	< 0.05	50	Serious	Serious	No	No	Serious	Very low
	Liu H 2020 (39)	MD: 0.12 (0.05, 0.18)	< 0.05	88	Serious	Serious	No	No	Serious	Very low
	Chen F 2021 (40)	MD: 0.08 (0.05, 0.11)	< 0.001	50.2	Serious	Serious	No	No	Serious	Very low
BGP	Li 2020 J (29)	MD: -1.10 (-1.63, -0.57)	< 0.001	0	Serious	No	No	No	Serious	Low
	Chen T 2020 (30)	MD: 1.86 (1.11, 2.61)	< 0.01	86	Serious	Serious	No	Serious	Serious	Very low
	Chen P 2021 (32)	MD: -2.89 (-4.60, -1.17)	0.001	98	Serious	Serious	No	Serious	Serious	Very low
	Cai X 2022 (35)	MD: 2.13 (1.77, 2.49)	< 0.001	42	Serious	No	No	Serious	Serious	Very low
ALP	Chen T 2020 (30)	MD: -7.78 (-17.0, 1.43)	0.1	97	Serious	Serious	No	Serious	Serious	Very low
	Zhao S 2019 (31)	SMD: 0.22 (-0.68, 0.25)	0.361	71.6	Serious	Serious	No	No	Serious	Very low
	Ma X 2022 (33)	MD: -17.63 (-26.01, -9.25)	< 0.001	73	Serious	Serious	No	Serious	Serious	Very low
	Cai X 2022 (35)	MD: -3.02 (-4.46, -1.59)	< 0.001	0	Serious	No	No	No	Serious	Low
	Zhang Y 2021 (37)	MD: -3.56 (-5.98, -1.15)	0.004	0	Serious	No	No	No	Serious	Low
	An Y 2019 (42)	MD: -1.48 (-3.24, 0.29)	0.10	0	Serious	No	No	No	Serious	Low
S-Ca	Chen T 2020 (30)	MD: 0.07 (-0.05, 0.19)	0.23	78	Serious	Serious	No	No	Serious	Very low
	Zhao S 2019 (31)	SMD: -0.05 (-0.09, 0.00)	0.033	66.8	Serious	Serious	No	No	Serious	Very low
	Cai X 2022 (35)	MD: 0.26 (0.19, 0.33)	< 0.001	0	Serious	No	No	No	Serious	Low
	Jing Y 2021 (36)	MD: 0.22 (0.17, 0.27)	< 0.001	49	Serious	No	No	No	Serious	Low
	Zhang Y 2021 (37)	MD: 0.21 (0.17, 0.25)	< 0.001	91	Serious	Serious	No	No	Serious	Very low
	An Y 2019 (42)	MD: -0.03 (-0.07, 0.01)	0.10	35	Serious	No	No	No	Serious	Low
S-P	Zhao S 2019 (31)	SMD: -0.02 (-0.11, 0.07)	0.639	49.3	Serious	No	No	No	Serious	Low
	Cai X 2022 (35)	MD: 0.05 (0.00, 0.10)	0.05	0	Serious	No	No	No	Serious	Low
	Zhang Y 2021 (37)	MD: -0.22 (-0.29, -0.15)	< 0.001	0	Serious	No	No	No	Serious	Low
	An Y 2019 (42)	MD: 0.02 (-0.02, 0.06)	0.36	47	Serious	No	No	No	Serious	Low
E2	Li 2020 J (29)	SMD: 0.49 (0.30, 0.68)	< 0.001	6	Serious	No	No	No	Serious	Low
	Chen T 2020 (30)	MD: 7.20 (2. 82, 11.58)	0.001	98	Serious	Serious	No	Serious	Serious	Very low
	Zhao S 2019 (31)	SMD: 0.62 (0.28, 1.52)	0.177	93.9	Serious	Serious	No	No	Serious	Very low
	Ma X 2022 (33)	MD: 3.15 (0.28, 6.02)	0.03	62	Serious	Serious	No	Serious	Serious	Very low
	Cai X 2022 (35)	MD: 7.03 (3.29, 10.77)	< 0.001	99	Serious	Serious	No	Serious	Serious	Very low
	Jing Y 2021 (36)	MD: -0.58 (-1.54, 0.38)	0.24	0	Serious	No	No	No	Serious	Low
	Zhang Y 2021 (37)	MD: 6.72 (4.96, 8.47)	< 0.001	0	Serious	No	No	No	Serious	Low
	Chen F 2021 (40)	MD: -0.44 (-4.95, 4.06)	0.847	0	Serious	No	No	No	Serious	Low
IL-6	Zhao S 2019 (31)	SMD: -1.78 (-4.86, 1.30)	0.258	98.2	Serious	Serious	No	Serious	Serious	Very low
	Ma X 2022 (33)	MD: -5.72 (-14.05, 2.61)	0.18	73	Serious	Serious	No	Serious	Serious	Very low
Clinical Effective Rate	Li 2020 J (29)	OR: 5.07 (3.07, 8.35)	< 0.001	0	Serious	No	No	Serious	Serious	Very low
	Chen T 2020 (30)	OR: 3.84 (2.81, 5.23)	< 0.01	18	Serious	No	No	No	Serious	Low
	Zhao S 2019 (31)	RR: 1.35 (1.17, 1.54)	< 0.001	72.5	Serious	Serious	No	No	Serious	Very low
	Chen P 2021 (32)	OR: 3.45 (2.23, 5.33)	< 0.001	0	Serious	No	No	No	Serious	Low
	Ma X 2022 (33)	OR: 2.57 (1.37, 4.83)	0.003	0	Serious	No	No	No	Serious	Low
	Cui X 2019 (34)	OR: 4.66 (1.7, 12.81)	0.003	56	Serious	Serious	No	Serious	Serious	Very low
	Cai X 2022 (35)	OR: 7.25 (4.38, 11.99)	< 0.001	0	Serious	No	No	Serious	Serious	Very low
	Jing Y 2021 (36)	RR: 1.05 (0.97, 1.13)	0.25	22	Serious	No	No	No	Serious	Low
	Zhang Y 2021 (37)	OR: 3.56 (2.37, 5.35)	< 0.001	0	Serious	No	No	No	Serious	Low
	Wang Y 2020 (38)	OR: 3.81 (2.35, 6.44)	< 0.001	37	Serious	No	No	Serious	Serious	Very low
	Chen F 2021 (40)	RR: 1.90 (1.20, 3.01)	0.006	89.3	Serious	Serious	No	No	Serious	Very low
	Xu G 2021 (41)	RR: 1.29 (1.19, 1.39)	< 0.001	0	Serious	No	No	No	Serious	Low
	An Y 2019 (42)	OR: 0.97 (0.39, 2.39)	0.94	0	Serious	No	No	No	Serious	Low
VAS	Chen T 2020 (30)	MD: -0.92 (-1.03, -0.82)	< 0.01	97	Serious	Serious	No	No	Serious	Very low
	Zhao S 2019 (31)	SMD: 0.55 (-1.03, 2.13)	0.496	97	Serious	Serious	No	No	Serious	Very low
	Chen P 2021 (32)	MD: -0.88 (-1.71, -0.06)	0.04	89	Serious	Serious	No	No	Serious	Very low
	Cui X 2019 (34)	MD: -1.15 (-1.77, -0.53)	< 0.001	63	Serious	Serious	No	No	Serious	Very low
	Wang Y 2020 (38)	MD: -0.50 (-0.77, -0.22)	0.004	0	Serious	No	No	No	Serious	Low
	Liu H 2020 (39)	MD: -1.44 (-1.70, -1.18)	< 0.05	0	Serious	No	No	No	No	Moderate
	Chen F 2021 (40)	MD: -1.05 (-1.32, -0.78)	< 0.001	29.4	Serious	No	No	No	Serious	Low
	An Y 2019 (42)	MD: -1.71 (-2.39, -1.03)	< 0.001	58	Serious	Serious	No	No	Serious	Very low
AE	Chen P 2021 (32)	OR: 0.24 (0.09, 0.63)	0.004	0	Serious	No	No	No	Serious	Low
	Liu H 2020 (39)	OR: 1.42 (0.77, 2.62)	0.27	0	Serious	No	No	No	Serious	Low
	Xu G 2021 (41)	RR: 0.65 (0.35, 1.20)	0.17	0	Serious	No	No	No	Serious	Low

ALP, Alkaline Phosphatase; E2, Estradiol; S-Ca, Serum Ca; S-P, Serum Phosphorus; BGP, Bone Gla Protein; VAS, Visual Analogue Scale; AE, Adverse Event; BMD, Bone Mineral Density; LBMD, BMD of Lumbar Spine; FTBMD, BMD of Femoral Trochanter; FNBMD, BMD of Femoral Neck; HBMD, BMD of Hip; MD, weighted mean difference; SMD, standard mean difference; OR, odds ratio; RR, risk ratio; CI, confidence intervals.

**Table 4:** Quality of Evidence in the included systematic reviews or Meta-analysis.

Outcomes	Level of Evidence *n*(%)			
	Low	Very Low	Moderate	High
LBMD	8 (57)	6 (43)	0 (0)	0 (0)
FNBMD	5 (63)	3 (37)	0 (0)	0 (0)
HBMD	1 (33)	2 (67)	0 (0)	0 (0)
FTBMD	3 (75)	1 (25)	0 (0)	0 (0)
Ward’s triangle BMD	0 (0)	3 (100)	0 (0)	0 (0)
BGP	1 (25)	3 (75)	0 (0)	0 (0)
ALP	3 (50)	3(50)	0 (0)	0 (0)
S-Ca	3 (50)	3(50)	0 (0)	0 (0)
S-P	4 (100)	0 (0)	0 (0)	0 (0)
E2	4 (50)	4 (50)	0 (0)	0 (0)
IL-6	0 (0)	2 (100)	0 (0)	0 (0)
Clinical Effective Rate	7 (54)	6 (46)	0 (0)	0 (0)
VAS	2 (24)	5 (63)	1 (13)	0 (0)
AE	3 (100)	0 (0)	0 (0)	0 (0)
Total	44 (51)	41 (48)	1 (1)	0 (0)

ALP, Alkaline Phosphatase; E2, Estradiol; S-Ca, Serum Ca; S-P, Serum Phosphorus; BGP, Bone Gla Protein; VAS, Visual Analogue Scale; AE, Adverse Event; BMD, Bone Mineral Density; LBMD, BMD of Lumbar Spine; FTBMD, BMD of Femoral Trochanter; FNBMD, BMD of Femoral Neck; HBMD, BMD of Hip.

### 3.4 Outcome indicators

#### 3.4.1 BMD

##### 3.4.1.1 BMD of the lumbar spine (LBMD)

The LBMD data were combined and analysed in the 14 included studies. In terms of improving the LBMD, the conclusions of 9 studies (29–32, 35, 37, 40–42) indicated that the treatment administered in the EG had a better curative effect than that administered in the CG (mean difference (MD): 0.04 to 0.88), while the other 5 studies (33, 34, 36, 38, 39) showed no difference between the two groups.

##### 3.4.1.2 BMD of the femoral neck (FNBMD)

The results of five studies (35, 37–39, 41) indicated that the treatment administered in the EG improved the FNBMD better than that administered in the CG (MD: 0.06 to 0.89), with a statistically significant difference (*P* < 0.05). However, three studies (31, 33, 40) found that there was no significant difference between the two groups.

##### 3.4.1.3 BMD of the hip (HBMD)

Three studies (36, 38, 42) reported the combined effect on the HBMD. In two studies (36, 38), researchers believed that the treatment administered in the EG could improve the HBMD better than that administered in the CG, while there was no difference between the two groups in one study (MD= 0.02, 95% CI: -0.01 to 0.05, *P* = 0.26) (33).

##### 3.4.1.4 BMD of the femoral trochanter (FTBMD)

In three studies (37, 40, 41), researchers believed that the treatment administered in the EG could improve the FTBMD better than that administered in the CG, while the results of one study (39) showed that there was no significant difference between the EG and the CG in terms of FTBMD improvement (MD = 0.03, 95% CI: 0.00 to 0.07, *P* = 0.07).

##### 3.4.1.5 BMD of Ward’s triangle

Three studies (37, 39, 40) showed the combined effect on the BMD of Ward’s triangle, and the results of these three studies suggest that YSHZT can improve the BMD of Ward’s triangle better than a conventional drug treatment or placebo (MD: 0.03 to 0.12); however, the level of evidence is very low.

#### 3.4.2 Serum indicators

##### 3.4.2.1 Bone Gla protein (BGP)

Researchers in four studies (29, 30, 32, 35) conducted a combined analysis of BGP. The results of two studies (30, 35) indicated that the treatment administered in the EG increased the BGP level more than the treatment administered in the CG, while the results of the other two studies (29, 32) were the opposite, with a statistically significant difference (*P* < 0.01).

##### 3.4.2.2 ALP

The researchers in three studies (33, 35, 37) believed that YSHZT had a worse effect on increasing the ALP level than the treatment administered in the CG (MD: -17.63 to -3.02), with a statistically significant difference (*P* < 0.001). In another three studies (30, 31, 42), there was no significant difference in the increase in the ALP level between the two groups.

##### 3.4.2.3 S-Ca

In three studies (35–37), the treatment provided in the EG was believed to increase the S-Ca content more than the treatment provided in the CG, with a statistically significant difference (*P* < 0.001). There were also three studies (30, 31, 42) in which there was no significant difference between the two groups in terms of S-Ca level increase.

##### 3.4.2.4 S-P

One study (37) showed that the effect of increasing the S-P level in the EG was worse than that in the CG (MD= -0.22, 95% CI: -0.29 to -0.15, *P* < 0.001), while researchers in three studies (31, 35, 42) believed that there was no difference between the two groups in terms of the effect on the S-P level.

##### 3.4.2.5 E2

Six studies (29–31, 33, 35, 37) showed that YSHZT could significantly increase the E2 level in PMOP patients, while two studies (36, 40) showed that there was no difference between the two groups (*P* > 0.05).

##### 3.4.2.6 IL-6

Two studies (31, 33) showed that there was no significant difference in the reduction of the serum IL-6 level between YSHZT and conventional drugs or a placebo (*P* > 0.05), and the level of evidence was very low.

#### 3.4.3 Other indicators

##### 3.4.3.1 Clinical effective rate

A total of 13 studies reported the effective rate; among them, 11 studies (29–35, 37, 38, 40, 41) showed better efficacy for the treatment administered in the EG than that administered in the CG (*P* < 0.05) in the treatment of PMOP, while the other two studies (36, 42) showed no difference between the two groups.

##### 3.4.3.2 VAS score

The comparison of the VAS score indicated that, according to the results of seven studies (30, 32, 34, 38–40, 42), YSHZT could lead to lower VAS scores than conventional drugs or a placebo (MD: -1.71 to -0.50). Among them, one study (39) had a moderate level of evidence, and the remaining studies had a low or very low level of evidence.

#### 3.4.4 AEs

Two studies (39, 41) reported no difference in AEs between the EG and the CG. One SR (32) concluded that the adverse reaction rate in the YSHZT group was lower than that in the CG (OR: 0.24, 95% CI: 0.09 to 0.63, *P* = 0.004).

## 4 Discussion

In TCM, PMOP belongs to the categories of *Guwei* and *Gubi*, which are closely related to *kidney qi* weakness. On the other hand, because postmenopausal women are prone to blood stasis due to *liver qi* stagnation, it is particularly important to promote blood circulation. Based on the understanding of PMOP within the context of TCM, treating PMOP with YSHZT has attracted much attention. In this study, we included 14 SRs or MAs to evaluate the efficacy and safety of YSHZT for patients with PMOP. This study found that YSHZT can be applied to improve the BMD of Ward’s triangle. In addition, this review found that different SRs reported opposite conclusions regarding the outcome indicators of YSHZT in the treatment of PMOP, which indicates that the efficacy of YSHZT in PMOP is still uncertain. Among the SRs reporting a positive effect on improving the LBMD, the MD ranged from 0.04 to 0.88. In terms of improving bone transformation markers, YSHZT can significantly affect the serum BGP level. However, this review found that YSHZT can increase or decrease the serum BGP level, and the reason for this difference still needs further study. In the comparison of the effect on the serum ALP level, the MD of YSHZT in reducing the ALP level ranged from -17.63 to -3.02. The improvement of curative effects is an area of great concern in TCM. This review found that YSHZT can reduce the pain of PMOP patients, according to the conclusions of multiple SRs included in this review, with the MD ranging from -1.71 to -0.50. In terms of the comparison of AE rates, this review found that YSHZT has fewer side effects than the control or no significant difference compared with the control, which indicates that the application of YSHZT is safe. The findings of this study provide an evidence-based reference for the treatment of PMOP with YSHZT, which in turn provides a basis for further clinical application and research design.

### 4.1 Low methodological quality

The AMSTAR-2 tool was used to evaluate the methodological quality of all the included SRs or MAs, and the results showed that the methodological quality of the included literature was low, including 9 and 5 studies with low- and very low-level methodological quality. The reasons for the low-quality methodology of the SRs or MAs regarding the treatment of PMOP with YSHZT are summarized as follows: 1) none of the published studies provided a preliminary plan, which reduced their credibility; 2) two studies did not provide a literature exclusion list, which made it impossible for users to rule out the existence of selective bias; 3) none of the studies explained the source of funds for the study, which did not allow users to rule out potential conflicts of interest and thus affected the reliability of the conclusions; 4) one SR did not apply the correct data consolidation model, which reduced the reliability of the conclusion; 5) researchers in five SRs failed to fully investigate the possibility of publication bias, which may have affected the authenticity of the research results; 6) some SRs did not satisfactorily explain or discuss the source or treatment of heterogeneity; and 7) researchers in only one of the 14 SRs described potential conflicts of interest, which may have affected users’ confidence in their results. The SR/MA research plan for the treatment of PMOP with YSHZT needs to be improved with regard to the above defects to improve the methodological quality of SRs/MAs.

### 4.2 Weak level of evidence

Although the GRADE results of the evidence quality show that there is no high-level evidence regarding the use of YSHZT for PMOP, there is only 1 study with moderate evidence (1%), 44 studies with low-level evidence (51%) and 41 studies with very low-level evidence (48%), which indicates that the real efficacy of YSHZT may be very different from the estimated efficacy, and further research is likely to change the evaluation results. The reasons for the degradation are as follows: 1) the RCTs involved in the literature have great defects in terms of the randomization method, allocation concealment and blinding method, which affect the authenticity of the results; 2) the heterogeneity of some outcome indicators directly leads to the degradation of evidence quality, which is needed to further clarify the inclusion and exclusion criteria and to conduct appropriate subgroup analysis; and 3) some studies included a small number of studies, and most of the research conclusions were positive results, which increased the bias of the estimated effect. This study shows that the level of clinical evidence regarding the relevant outcome indicators of YSHZT for PMOP is low; thus, future research is needed to further optimize the research design according to the above shortcomings and to select the correct statistical methods to provide higher quality evidence.

### 4.3 Limitations

The limitations of this study mainly include the following: 1) only one study in English is included, while the rest are in Chinese; 2) TCM therapies that involve YSHZT are dissimilar, which may affect the extrapolation and application of the conclusion; and 3) the methodological quality and evidence level of studies included in this systematic evaluation are low, which reduces the reliability of the research results.

## 5 Conclusions

In summary, because the methodological quality and the level of evidence of YSHZT for PMOP are poor, clinicians and decision makers are reminded to use this combination of evidence in specific situations. In the future, it is necessary to conduct large-sample, high-quality and long-term research studies to further evaluate the efficacy and safety of YSHZT for PMOP. Original studies, such as RCTs, should be designed, implemented and reported in strict accordance with clinical trial quality management norms and other relevant norms. In addition, researchers conducting SRs or MAs on the use of YSHZT for PMOP should strictly follow the AMSTAR-2 tool and the PRISMA statement to provide higher quality evidence-based medicine.

## Data availability statement

The original contributions presented in the study are included in the article/**Supplementary Material**. Further inquiries can be directed to the corresponding author.

## Author contributions

JZ and XX conceptualised the idea and planned the study. JZ led on running literature searches, critical appraisal of the literature, and writing of the manuscript. GZ acted as a second independent searcher and assisted with critical appraisal and manuscript preparation. NX assisted with manuscript preparation. JL and JZ provided key edits and the final version of the manuscript. JL provided overall supervision of the work. All authors contributed to the article and approved the submitted version.

## Funding

This work was supported by the National key research and development program (2021YFC1712804), the Natural Science Foundation of Guangdong Province (No. 2022A1515010385, No. 2022A1515011700), the National Natural Science Foundation of China (No. 81873314), the Project of Guangdong Provincial Department of Finance (No. [2018]8), Research Fund for Bajian Talents of Guangdong Provincial Hospital of Chinese Medicine (No. BJ2022KY01), Project of Philosophy and Social Science Planning of Guangzhou (No. 2022GZQN42) and the Science and Technology Research Project of the Second Affiliated Hospital of Guangzhou University of Chinese Medicine (YN2019ML08, YN2015MS15).

## Conflict of interest

The authors declare that the research was conducted in the absence of any commercial or financial relationships that could be construed as a potential conflict of interest.

## Publisher’s note

All claims expressed in this article are solely those of the authors and do not necessarily represent those of their affiliated organizations, or those of the publisher, the editors and the reviewers. Any product that may be evaluated in this article, or claim that may be made by its manufacturer, is not guaranteed or endorsed by the publisher.
