# Supplementary material for: Effectiveness of Yushen Hezhi therapy for postmenopausal osteoporosis: An overview of systematic reviews of randomized controlled trials
Source: Front Endocrinol (Lausanne). 2022 Sep 26;13:1015483. doi: 10.3389/fendo.2022.1015483 (PMC9548895; doi:10.3389/fendo.2022.1015483)
Supplement: Supplementary file 1 [file Table_1.docx]

**Supplementary material 1 Retrieval strategy.**

**Pubmed**

#1 (("bushen huoxue"[Supplementary Concept] OR "bushen huoxue"[All Fields]) AND ("therapeutics"[MeSH Terms] OR "therapeutics"[All Fields] OR "therapies"[All Fields] OR "therapy"[MeSH Subheading] OR "therapy"[All Fields] OR "therapy s"[All Fields] OR "therapys"[All Fields])) OR (("tonify"[All Fields] OR "tonifying"[All Fields]) AND ("kidney"[MeSH Terms] OR "kidney"[All Fields] OR "kidneys"[All Fields] OR "kidney s"[All Fields])) OR ("bushen huoxue"[Supplementary Concept] OR "bushen huoxue"[All Fields]) OR ("Bushen"[All Fields] AND "huayu"[All Fields]) OR ("Bushen"[All Fields] AND "quyu"[All Fields]) OR (("promote"[All Fields] OR "promoted"[All Fields] OR "promotes"[All Fields] OR "promoting"[All Fields] OR "promotion"[All Fields] OR "promotional"[All Fields] OR "promotions"[All Fields] OR "promotive"[All Fields]) AND ("blood circulation"[MeSH Terms] OR ("blood"[All Fields] AND "circulation"[All Fields]) OR "blood circulation"[All Fields])) OR ("chin med"[Journal] OR ("chinese"[All Fields] AND "medicine"[All Fields]) OR "chinese medicine"[All Fields]) OR (("asians"[MeSH Terms] OR "asians"[All Fields] OR "chinese"[All Fields] OR "chineses"[All Fields]) AND "Herb"[All Fields]) OR (("asians"[MeSH Terms] OR "asians"[All Fields] OR "chinese"[All Fields] OR "chineses"[All Fields]) AND ("herbal medicine"[MeSH Terms] OR ("herbal"[All Fields] AND "medicine"[All Fields]) OR "herbal medicine"[All Fields] OR "herbalism"[All Fields] OR "herbal"[All Fields] OR "herbals"[All Fields])) OR ("trends cardiovasc med"[Journal] OR "case manager"[Journal] OR "tcm"[All Fields])

#2 "osteoporosis, postmenopausal"[MeSH Terms] OR "Osteoporosis"[MeSH Terms] OR "Osteoporosis"[Title/Abstract] OR "postmenopausal osteoporosis"[Title/Abstract] OR "bone mineral density"[Title/Abstract] OR "bone density"[MeSH Terms] OR "PMOP"[Title/Abstract] OR "OP"[Title/Abstract]

#3 "systematic review"[Title/Abstract] OR "Meta-analysis"[Title/Abstract] OR "Meta-analysis"[Title/Abstract] OR "Meta-analysis"[Publication Type] OR "systematic review"[Publication Type]

#4 #1 AND #2 AND #3

**Embase**

#1 'osteoporosis'/exp OR osteoporosis OR 'postmenopausal osteoporosis':ab,ti OR 'bone mineral density':ab,ti OR pmop:ab,ti OR op:ab,ti

#2 'systematic review'/exp OR 'systematic review' OR 'systematic review':ab,ti OR 'meta analysis':ab,ti

#3 'chinese medicine'/exp OR 'chinese medicine' OR 'bushen huoxue therapy':ab,ti OR 'tonifying kidney':ab,ti OR 'bushen huoxue':ab,ti OR 'bushen huayu':ab,ti OR 'bushen quyu':ab,ti OR 'promoting blood circulation':ab,ti OR 'chinese herb':ab,ti OR 'chinese herbal':ab,ti OR tcm:ab,ti

#4 #1 AND #2 AND #3

**Cochrane Library**

#1 (Chinese Medicine) OR (Bushen Huoxue Therapy) OR (tonifying kidney) OR (Bushen huoxue) OR (Bushen huayu)

#2 (Bushen quyu) OR (promoting blood circulation) OR (Chinese Herb) OR (Chinese Herbal) OR (TCM)

#3 #1 OR #2

#4 (Osteoporosis):ti,ab,kw OR (Postmenopausal osteoporosis):ti,ab,kw OR (bone mineral density):ti,ab,kw OR (PMOP):ti,ab,kw OR (OP):ti,ab,kw

#5 (systematic review):ti,ab,kw OR ("meta-analysis"):ti,ab,kw OR ("meta analysis"):ti,ab,kw OR ("meta-analysis"):pt OR (systematic review):pt

#6 #3 AND #4 AND #5

**CNKI**

((Bushen Huoxue) or (Bushen) or (Huoxue) or (Huayu)) and ((Osteoporosis) or (Postmenopausal osteoporosis) or (one mineral density)) and ((systematic review) or (meta))

**WanFang**

((Bushen Huoxue) or (Bushen) or (Huoxue) or (Huayu)) and ((Osteoporosis) or (Postmenopausal osteoporosis) or (one mineral density)) and ((systematic review) or (meta))

**CBM**

((Bushen Huoxue) or (Bushen) or (Huoxue) or (Huayu)) and ((Osteoporosis) or (Postmenopausal osteoporosis) or (one mineral density)) and ((systematic review) or (meta))

**Supplementary material 2 A Measurement Tool to Assess Systematic Reviews (AMSTAR 2) items.**

1. Did the research questions and inclusion criteria for the review include the components of PICO?

2. Did the report of the review contain an explicit statement that the review methods were established prior to the conduct of the review and did the report justify any significant deviations from the protocol?

3. Did the review authors explain their selection of the study designs for inclusion in the review?

4. Did the review authors use a comprehensive literature search strategy?

5. Did the review authors perform study selection in duplicate?

6. Did the review authors perform data extraction in duplicate?

7. Did the review authors provide a list of excluded studies and justify the exclusions?

8. Did the review authors describe the included studies in adequate detail?

9. Did the review authors use a satisfactory technique for assessing the risk of bias in individual studies that were included in the review?

10. Did the review authors report on the sources of funding for the studies included in the review?

11. If meta-analysis was performed did the review authors use appropriate methods for statistical combination of results?

12. If meta-analysis was performed, did the review authors assess the potential impact of risk of bias in individual studies on the results of the meta-analysis or other evidence synthesis?

13. Did the review authors account for risk of bias in individual studies when interpreting/ discussing the results of the review?

14. Did the review authors provide a satisfactory explanation for, and discussion of, any heterogeneity observed in the results of the review?

15. If they performed quantitative synthesis did the review authors carry out an adequate investigation of publication bias (small study bias) and discuss its likely impact on the results of the review?

16. Did the review authors report any potential sources of conflict of interest, including any funding they received for conducting the review?
