# Supplementary material for: Effectiveness of Yushen Hezhi therapy for postmenopausal osteoporosis: An overview of systematic reviews of randomized controlled trials
Source: Front Endocrinol (Lausanne). 2022 Sep 26;13:1015483. doi: 10.3389/fendo.2022.1015483 (PMC9548895; doi:10.3389/fendo.2022.1015483)
Supplement: Supplementary file 2 [file Table_2.docx]

**Supplementary material 3 List of studies excluded after reading the full text, along with the reasons.**

| **Paper Title** | **Reasons for exclusion** |
| --- | --- |
| Efficacy and safety of Xianling Gubao capsule in treating postmenopausal osteoporosis: A protocol for systematic review and meta-analysis. | Protocol |
| Effect of downregulation of serum MMP-3 levels by traditional Chinese medicine ingredients combined with methotrexate on the progression of bone injury in patients with rheumatoid arthritis: A protocol for a systematic review and meta-analysis. |  |
| Comparison of efficacy and safety of Chinese patent medicine in the treatment of postmenopausal osteoporosis: A protocol for systematic review and network meta-analysis. |  |
| Tai Chi on bone mineral density of postmenopausal osteoporosis: A protocol for systematic review and meta-analysis. |  |
| Effectiveness and safety of Guilu Erxian Glue (a traditional Chinese medicinal product) for the treatment of postmenopausal osteoporosis: A protocol for systematic review and meta-analysis. |  |
| Effects of Gushukang for postmenopausal osteoporosis: A protocol for systematic review and meta-analysis. |  |
| Effects of bushen qianggu method for primary osteoporosis: A protocol for systematic review and meta-analysis. |  |
| Efficacy of Xianling Gubao capsule in treating sarco-osteopenia: Protocol for a systematic review and meta-analysis. |  |
| Efficacy and safety of Zuogui Pill in treating osteoporosis: Study protocol of a systematic review. |  |
| Acupuncture for primary osteoporosis: A network meta-analysis of randomized controlled trials protocol. |  |
| Liuwei Dihuang Decoction for primary osteoporosis: A protocol for a systematic review and meta-analysis. |  |
| Comparative Efficacy of Xianling Gubao Capsules in Improving Bone Mineral Density in Postmenopausal Osteoporosis: A Network Meta-Analysis. | Network meta-analysis |
| Pharmacological and non-pharmacological interventions for osteoporosis: A protocol for an overview with an evidence map and a network meta-analysis of trials. |  |
| Efficacy of Chinese patent medicine for primary osteoporosis: A network meta-analysis. |  |
| Effectiveness associated with different therapies for senile osteoporosis: a network Meta-analysis. |  |
| Acupuncture for primary osteoporosis: A network meta-analysis of randomized controlled trials protocol. |  |
| Efficacy and safety of oral kidney-tonifying Chinese patent medicines in treatment of postmenopausal osteoporosis: A network Meta-analysis. |  |
| Oral Chinese patent medicine containing Epimedium in treatment of postmenopausal osteoporosis: A bayesian network Meta-analysis |  |
| Efficacy of Jintiange Capsules in the Treatment of Osteoporosis: A Network Meta-analysis. |  |
| Bayesian Network Meta-analysis of Therapeutic Efficacy of Xianling Gubao Capsules Combined with Routine Therapy for Postmenopausal Osteoporosis |  |
| Network Meta-analysis of Four Kinds of Traditional Chinese Fitness Exercises in the Treatment of Osteoporosis in the Elderly |  |
| Acupuncture combined with other therapies for osteoporosis: a network meta-analysis |  |
| Network meta analysis of different intervention measures in the treatment of osteoporotic vertebral compression fracture |  |
| Network meta analysis of nursing effect of nursing style on senile osteoporosis fracture |  |
| Network meta-analysis of different drugs for the treatment of primary osteoporosis |  |
| Efficacy and safety of anti - osteoporosis drugs for the prevention of osteoporotic fractures in postmenopausal women: a network Meta-analysis |  |
| Network meta analysis of anti osteoporosis drugs in the treatment of glucocorticoid induced osteoporosis |  |
| Anti-osteoporosis Drugs Prevent Fracture Risk in Patients with Primary Osteoporosis: Network Meta-analysis Based on Bayesian Framework |  |
| Meta Analysis of Tonifying Kidney and Activating Blood Ointment in the Treatment of Primary Osteoporosis. | Not PMOP |
| Acupoint injection treatment for primary osteoporosis: A systematic review and meta-analysis of randomized controlled trials protocol. |  |
| Moxibustion therapy for treating patients with primary osteoporosis: A systematic review and meta-analysis protocol. |  |
| Liuwei Dihuang Decoction for primary osteoporosis: A protocol for a systematic review and meta-analysis. |  |
| Effects of Nonpharmacological Interventions on Balance Function in Patients with Osteoporosis or Osteopenia: A Network Meta-Analysis of Randomized Controlled Trials. |  |
| Efficacy of Jintiange Capsules in the Treatment of Osteoporosis: A Network Meta-analysis. |  |
| Comparison of Bone Mineral Density in Lumbar Spine and Fracture Rate among Eight Drugs in Treatments of Osteoporosis in Men: A Network Meta-Analysis. |  |
| A Meta-Analysis on the Efficacy of Tonic Herb on Osteoporosis |  |
| Meta analysis of the efficacy and safety of kidney tonifying and blood activating therapy in the treatment of diabetes osteoporosis |  |
| Curative effect of kindey-toinfying on bone loss associated with endocrine therapy in breast cancer : a Meta-analysis |  |
| Effectiveness of TCM Kidney Tonics in Improving Chronic Pain of Primary Osteoporosis: A Meta-analysis |  |
| Meta-analysis of invigorating kidney， invigorating spleen and promoting blood circulation principle in the treatment of middle-aged and elderly primary osteoporosis |  |
| Systematic evaluation of the clinical effect of Bushen Zhuanggu Decoction on primary osteoporosis |  |
| Bibliometric analysis of osteoporosis secondary to rheumatoid arthritis and meta-analysis of traditional Chinese medicine treatment |  |
| To explore the curative effect of Bushen Zhuanggu Decoction on osteoporosis based on systematic evaluation and network pharmacology |  |
| Meta analysis and trial sequential analysis of Bushen Huoxue preparation combined with calcium in the treatment of senile osteoporosis |  |
| A meta-analysis of efficacy of two kinds of the Bushen medicine on BMD and VAS in osteoporosis patients |  |
| A Meta-analysis on the Prevention and Treatment of Senile Osteoporosis with Traditional Chinese Medicine of Tonifying Liver and Kidney and Promoting Blood Circulation |  |
| Meta - analysis on clinical efficacy of primary osteoporosis treated with integrated Chinese and Western medicine |  |
| Meta-analysis of Bushen Jiangu Chinese medicine in treating osteoporosis |  |
| Meta analysis and clinical observation on the treatment of early-onset ovarian insufficiency with traditional Chinese medicine combined with hormone supplementation |  |
| Systematic evaluation and meta-analysis of Chinese herbal compound in preventing and treating osteoporosis related to endocrine therapy in breast cancer |  |
| Meta-analysis on Bushenzhuangjin Decoction in orthopaedics clinical application |  |
| Systematic Ｒeview of Randomized Controlled Trials on Kidney-tonifying Chinese Patent Medicine in Treatment of Senile Osteoporosis |  |
| Meta-analysis of the Curative Effect of Tonifying-kidney-to-strengthen-bone Method in the Treatment of Diabetic Osteoporosis |  |
| Meta-analysis of Reinforcing Kidney and Invigorating Spleen Principle in the Treatment of Primary Osteoporosis |  |
| Meta-analysis of Bushen Jianpi and Huoxue Huayu Chinese Medicine Compound Combined with Caltrate D3 in the Treatment of Primary Osteoporosis |  |
| Systematic evaluation of the therapeutic effect of kidney tonifying Chinese medicine on primary osteoporosis |  |
| Clinical study on the treatment of osteoporosis with Yang's Bushen Huoxue Decoction |  |
| The Effect of Selected Herbal Medicines on Bone Turnover Markers: A Systematic Review and Meta-Analysis. |  |
| Chinese Proprietary Medicine Xianling Gubao Capsule for Osteoporosis: A Systematic Review and Meta-Analysis of Randomized Clinical Trials. |  |
| Chinese patent medicine for osteoporosis: a systematic review and meta-analysis. |  |
| Efficacy and safety of Duhuo Jisheng Decoction add-on bisphosphonate medications in patients with osteoporosis: A meta-analysis of randomized controlled trials. |  |
| Different Training Durations and Frequencies of Tai Chi for Bone Mineral Density Improvement: A Systematic Review and Meta-Analysis. |  |
| The efficacy and safety of traditional Chinese medicine's tonifying-kidney, strengthening-spleen, and invigorating-blood circulation (Bushen-Jianpi-Huoxue) principle for type 2 diabetes mellitus with osteoporosis: A protocol for systematic review and meta-analysis. |  |
| Effectiveness of electroacupuncture as a treatment for osteoporosis: A systematic review and meta-analysis. |  |
| Benefits of Herbal Medicine on Bone Mineral Density in Osteoporosis: A Meta-Analysis of Randomized Controlled Trials. |  |
| The Efficacy and Safety of Traditional Chinese Medicine Tonifying-Shen (Kidney) Principle for Primary Osteoporosis: A Systematic Review and Meta-Analysis of Randomized Controlled Trials. |  |
| Effect of Tai Chi for the prevention or treatment of osteoporosis in elderly adults: protocol for a systematic review and meta-analysis. | Not based on YSHZT |
| Tuina for osteoporosis: A systematic review protocol. |  |
| Acupuncture for osteoporosis: a systematic review protocol. |  |
| Acupoint injection treatment for primary osteoporosis: A systematic review and meta-analysis of randomized controlled trials protocol. |  |
| Meta analysis on the treatment of postmenopausal osteoporosis with traditional Chinese medicine based on regulating liver and kidney |  |
| Meta - Analysis of the Efficacy and Safety of Combined Chinese and Western Medi- cine in the Treatment of Primary Osteoporosis |  |
| Efficacy and safety of Chinese medicine for Tonifying kidney in the treatment of postmenopausal osteoporosis: a systematic review and meta-analysis |  |
| Efficacy of Kidne y-tonifying Chine se patent medicine on bone turnover makers in postmenopausal oste oporosis: A me ta-analysis |  |
| Meta analysis of Chinese medicine in the treatment of primary osteoporosis and clinical observation of Gubao capsule |  |
| Warming Kidney Decoction in the Treatment of Postmenopausal Osteoporosis: A Systematic Ｒeview and Meta-analysis of Ｒandomized Controlled Trials |  |
| Meta analysis of the effect of kidney tonifying therapy on aromatase inhibitor related bone loss |  |
| Clinical observation of alendronate sodium and Bushen Zhuanggu oral liquid in the treatment of postmenopausal osteoporosis |  |
| Systematic evaluation on the treatment of primary osteoporosis by oral administration of traditional Chinese Medicine |  |
| Systematic evaluation of the efficacy and safety of traditional Chinese medicine in treating postmenopausal osteoporosis |  |
| Meta analysis of the effect of kidney tonifying herbs on bone mineral density in postmenopausal osteoporosis patients |  |
| Meta-analysis on the treatment of osteoporotic vertebral compression fractures with traditional Chinese medicine combined with PKP | OP fracture |
| Meta analysis of the curative effect of kidney tonifying, blood activating and bone strengthening therapy combined with PFNA on osteoporotic intertrochanteric fracture of femur |  |
| Meta-analysis of the effect of Bushen Zhuanggu Decoction on vertebroplasty in patients with osteoporotic thoracolumbar  compression fracture |  |
| Meta-analysis of Bushen Jiangu Decoction in the Treatment of Vertebral Compression Fractures in Patients with Osteoporosis |  |
| Percutaneous vertebroplasty combined with nourishing kidney and strong bone decoction for the treatment of osteoporotic thoracolumbar vertebral compression fractures: a meta-analysis |  |
| Clinical observation on treating elderly osteoporotic spinal compression fracture by PKP plus the Bushen Zhuanggu decoction |  |
| Clinical Efficacy and Safety of Zoledronic Acid Combined with PVP/PKP in the Treatment of Osteoporotic Vertebral Compression Fracture: A Systematic Review and Meta-Analysis of Randomized Controlled Trials. |  |
| Meta-analysis of Taohong Siwu Decoction Assisted by Percutaneous Vertebral Augmentation for Osteoporotic Thoracolumbar Compression Fractures |  |
| Efficacy of Xianlinggubao Capsule in Patients of Osteoporotic Fractures: A Meta-Analysis |  |
